# Research on Variable Parameter Drilling Method of Ti-CFRP-Ti Laminated Stacks Based on Real-Time Sensing of Drilling Axial Force

**DOI:** 10.3390/s22031188

**Published:** 2022-02-04

**Authors:** Zhengzhu Zhang, Ning Zhang, Fenghe Wu, Weixiang Teng, Yingbing Sun, Baosu Guo

**Affiliations:** 1 College of Mechanical Engineering, Yanshan University, Qinhuangdao 066004, China; zhangzhengzhu@stumail.ysu.edu.cn (Z.Z.); niza@stumail.ysu.edu.cn (N.Z.); twx@stumail.ysu.edu.cn (W.T.); sunyingbing@ysu.edu.cn (Y.S.); guobaosu@ysu.edu.cn (B.G.); 2 Geely Holding Group, Hangzhou 310051, China; 3 Heavy-Duty Intelligent Manufacturing Equipment Innovation Center of Hebei Province, Qinhuangdao 066004, China

**Keywords:** intelligent tool holder system, Ti-CFRP-Ti laminated stacks, compressive sensing, drilling parameter optimization

## Abstract

Ti-CFRP-Ti laminated stacks have been widely used in aviation, aerospace, shipbuilding and other industries, owing to its excellent physical and electrochemical properties. However, chip blockages occur easily when drilling into Ti-CFRP-Ti laminated stacks, resulting in a rapid rise of drilling temperature and an increase of axial drilling force, which may lead to the intensification of tool wear and a decline of drilling quality. Cutting force signals can effectively reflect the drilling process and tool condition, however, the traditional plate dynamometer is typically difficult in realizing the follow-up online measurement. Therefore, an intelligent tool holder system for real-time sensing of the cutting force is developed and constructed in this paper, and the variable parameter drilling method of Ti-CFRP-Ti laminated stacks is studied on this basis. Firstly, an intelligent tool holder system with high flexibility and adaptability is designed; Secondly, a cutting force signal processing method based on compressed sensing (CS) theory is proposed to solve the problem of high-frequency signal transmission; Lastly, the drilling experiment of Ti-CFRP-Ti laminated stacks is carried out based on the intelligent tool holder system, and the drilling parameters are optimized using a compromise programming approach and analytic hierarchy process (AHP). The comparison of results show that the optimized drilling parameters can effectively reduce the hole wall surface roughness and improve the drilling efficiency while ensuring a small axial force.

## 1. Introduction

In recent years, the requirements for strength, stiffness, fatigue resistance and lightweight properties of materials are becoming more urgent, with the continuous progression of aerospace technology. Under this context, lightweight high-strength materials such as Carbon Fiber Reinforced Plastics (CFRP) and titanium alloy (Ti) have found widespread use in the field of aerospace [1,2]. In practical applications, titanium alloy and CFRP are usually mechanically connected to form laminated stacks, such as Ti-CFRP-Ti, which takes into account strength, wear resistance, corrosion resistance and electrochemical compatibility. It has subsequently been used widely in aerospace, marine, automotive industries and aircraft wing manufacturing [3,4,5].

Laminated stacks are generally assembled by using bolts or riveting, and consequently, a large number of installation holes need to be processed. In actual production, the laminated stacks are usually clamped as a whole and drilled at one time to ensure assembly accuracy and to improve the efficiency of processing [6,7]. However, there exist great differences between titanium alloy and CFRP in terms of machinability, physical and chemical properties, tool wear mechanisms and drilling parameter selection, resulting in the decline of drilling quality [8]. When drilling into Ti-CFRP-Ti laminate, the cutting tool encounters significant friction with the carbon fiber. Due to the low thermal conductivity of titanium alloy and the accumulation of cutting heat, the tool wear is severe and the service life decreases rapidly. In addition, titanium alloy material adheres to the rake face of the tool to form chip nodules, which in turn causes machining defects such as hole wall scratches, thus greatly affecting the drilling quality [9,10]. Therefore, improving the drilling process of Ti-CFRP-Ti laminated stacks is of great significance to ensure drilling quality and enhancing the service life of the cutting tool.

Due to the differences in physical and cutting properties between the CFRP and titanium alloy, the optimal cutting parameter range remains inconsistent. For example, CFRP is suitable for a high cutting speed and low feed rate, while titanium alloy recommends a low cutting speed and high feed rate [11,12,13]. Therefore, considering the factors such as drilling quality, machining efficiency and tool wear, researchers have attempted to promote the drilling effect of Ti-CFRP-Ti laminated stacks by optimizing and improving cutting parameters. Wang et al. [14] studied the influence of drilling parameters on the axial force, drilling temperature, and drilling quality in CFRP-aluminum laminate structures by using double-top diamond coated drills. The results showed that the axial force increases linearly with the increase in feed rate, and the cutting force during drilling notably affects burrs and stratification. Hu et al. [15] introduced a simulated annealing algorithm to optimize spindle speed and feed speed from the perspective of machining energy consumption (MEC), and the optimization effect was verified. At the same time, the influence of MEC minimization on processing efficiency was discussed, the results revealed that MEC minimization may lead to an increase of machining time. Feito et al. [16] constructed the relationship between cutting parameters and the axial force of composites through the response surface method, and multi-objective optimization of cutting parameters was carried out. Moreover, an artificial neural network model was constructed, which receives tool wear, spindle speed, feed rate and point-angle as inputs for cutting force predictions and drilling parameter optimization, with results showing that the thrust force is more sensitive to feed rate, and a low point-angle can avoid damaging the CFRP laminate [17]. The drilling axial force is extremely important for reflecting on the drilling process and evaluating the drilling quality. However, a majority of the current literature is based on employing a plate dynamometer for cutting force measurement, which is usually limited by its difficult installation and poor flexibility [18]. Thus, it remains necessary to design a follow-up real-time cutting force measurement system with high flexibility, high adaptability and high precision to improve the drilling process of Ti-CFRP-Ti laminated stacks.

Therefore, from the perspective of developing an intelligent hardware with a cutting force real-time sensing function, the overall integration scheme of a cutting force measurement system based on a tool holder is firstly studied. Secondly, a cutting force signal processing method based on compressive sensing is proposed. Lastly, the performance of a verification experiment on the intelligent tool holder system and a drilling experiment of Ti-CFRP-Ti laminated stacks are designed, and the multi-objective optimization of drilling parameters is realized based on the experimental results.

The remainder of this paper is organized as follow: in Section 2, the variable parameter drilling process of Ti-CFRP-Ti laminated stacks is analyzed. Then, in Section 3, the design idea of an intelligent tool holder system is proposed, including the overall system composition and cutting force signal processing method. Subsequently, in Section 4, the experimental system is built and the experimental scheme is designed. An analysis of the experimental results and optimization of drilling parameters are presented in Section 5. Finally, conclusions are provided in Section 6.

## 2. Analysis of Drilling Process for Ti-CFRP-Ti Laminated Stacks

**Figure 1:**
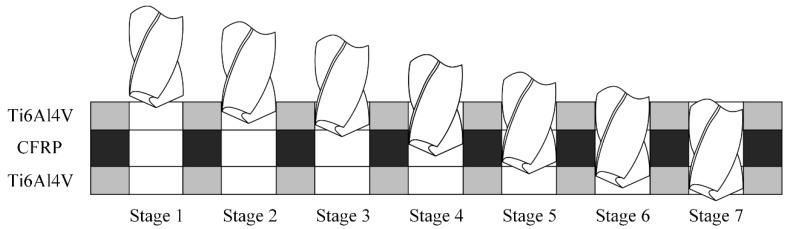
Seven stages of drilling Ti-CFRP-Ti laminated stacks.

Generally, a higher spindle speed and lower feed rate are used for drilling CFRP, while a lower spindle speed and higher feed rate are suitable for drilling titanium alloy. Therefore, it is necessary to select appropriate cutting parameters according to the mechanical properties of each material, which means that three groups of cutting parameters should be used for drilling three layers of materials, respectively. Variable parameter technology refers to the change of appropriate cutting parameters when drilling into the interface of two materials. According to the contact between the cutting tool and a workpiece material, the drilling process can be divided into seven stages, as shown in Figure 1. Stages 1 and 7 indicates that the drilling tool cut into the upper titanium alloy and cut out the lower titanium alloy, respectively. Stages 2, 4 and 6 displays the drilling of the upper titanium alloy, CFRP and the lower titanium alloy completely. Stages 3 and 5 are defined as the drilling of interface regions. In order to adapt the drilling parameters to different materials, the cutting parameters need to be changed in Stages 3 and 5. In order to obtain the reasonable parameters for the different layers, in this paper, the drilling parameters are optimized based on the drilling axial force information and the factors such as surface roughness of the hole wall and processing efficiency.

## 3. Design of Intelligent Tool Holder System

### 3.1. Overall System Composition

**Figure 2:**
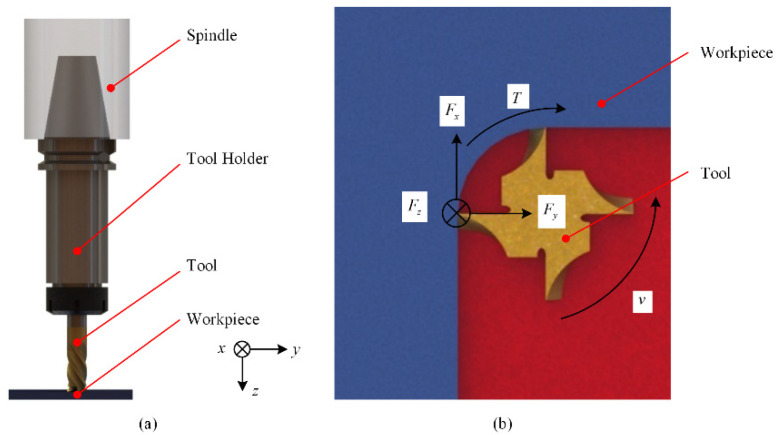
(**a**) composition of the cutting system; (**b**) cutting force on tool tip.

During the cutting process, the cutting system is composed of a spindle, tool holder, cutting tool and workpiece, as shown in Figure 2. Considering that the cutting force components and the torque generated in milling process are *F_x_*, *F_y_*, *F_z_* and *T*, as shown in Figure 2, and *F_z_* and *T* in drilling process, therefore, the system designed in this paper provides data for drilling Ti-CFRP-Ti laminated stacks by sensing the axial force *F_z_* and torque *T*. In order to realize the real-time perception of cutting force, it is necessary to study the high-precision force sensor, the optimization design of tool holder matrix structure and the signal processing transmission mode.

It was found from the author’s previous research [19] that the sensitivity of the axial force was lower when using a resistance strain sensor. Therefore, in this paper, the semiconductor strain gauge type SB5-350-P-2-X30 is selected for the axial force measured in the system designed while the resistance strain gauges type BF350-4HA-E (11)-N4 (Avic Zhonghang Electronic Measuring Instruments Co., Ltd., Xi’an, China) is selected for the torque, and the sensor bridge is constructed by means of a full Wheatstone bridge circuit. Moreover, a lithium battery is used to supply power to the whole system, and the power supply voltage is converted from 3.7 V to the required 3.3 V through a DC–DC circuit. The size of the encapsulated power supply module is 56 mm × 38 mm × 21 mm. A commercial tool holder type BT40-ER32-150L (Harbin Measuring Tools & Cutting Tools Group Co., Ltd., Harbin, China) is selected to integrate components such as resistance sensors, signal processing modules and power modules, thus the standard tool holder needs to be a secondary structure designed with consideration of the following requirements and characteristics.

(1) Universality. The basic structure of the improved tool holder enables the clamping of the tool holder system on the machine tool spindle and the clamping of the tool as well as the handling of the manipulator without changing the existing device of the cutting system.

(2) Dynamic balance. The centroid of the improved tool holder should be on the axis of rotation to avoid eccentricity and accidents.

(3) High sensitivity. As the elastic element, the improved tool holder should have good sensitivity to realize precise sensing of the micro cutting force.

(4) High stiffness. In order to ensure the machining accuracy in the cutting process, the improved tool holder should have good rigidity to reduce the cutting deformation.

(5) Manufacturability. The structure of the improved tool holder should be designed and fabricated on the premise of easy processing compared with the standard tool holder.

**Figure 3:**
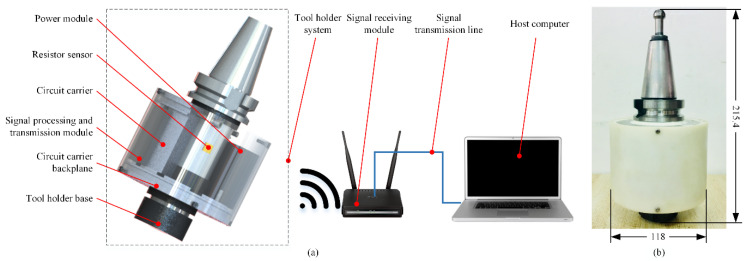
(**a**) intelligent tool holder system composition; (**b**) assembled system.

Following the above design criteria, it was determined that an annular groove is processed on the standard tool holder for attachment of the strain gauge to form a resistance sensor, and the circuit carrier is mounted outside the cylindrical surface of the tool holder, where two sets of signal processing modules and power modules are embedded on. The force data are collected and transmitted to the host computer via Wi-Fi. The system model and the assembled prototype are shown in Figure 3.

### 3.2. Cutting Force Signal Processing

The intelligent tool holder system has a high cutting frequency in the case of high-speed and multi-edge cutting. According to the classical Nyquist–Shannon sampling theorem, the sampling rate must be greater than twice the signal bandwidth to ensure accurate signal reconstruction. The maximum frequency is equal to the cutting frequency during the cutting process. This method generates redundant data and causes data congestion problems, due to the limitation of data bandwidth by the standard transmission protocols and hardware devices. By using prior knowledge of signal sparsity, compressive sensing (CS) theory indicates that the signal can be compressed while sampling, which achieves a sampling frequency far lower than Nyquist rate and sparse signals are reconstructed with high precision. Therefore, in order to realize the processing of cutting force signals based on CS, the sparse representation of cutting force signals, the acquisition of sampling signals, and the high-precision reconstruction of sampling signals are studied in this paper.

#### 3.2.1. Sparse Representation Model Construction

The sparse signal is defined when only a few elements are non-zero. However, few signals in nature are sparse in the time domain, and most signals may be sparse in the transform domain. According to the theory of harmonic analysis, the one-dimensional discrete signal ***x*** can be represented by a linear combination of a set of *N* standard bases that is arbitrary and the length is *N*. As follows in Equation (1):

(1) $$x=\sum_{i=1}^{N}\psi_{i}\cdot \alpha_{i}=\Psi \cdot \alpha$$

where Ψ is a *N* × *N* representation basis with $\psi_{i}$ as columns (sparse matrix), and α is a *N* × 1 sparse vector with $\alpha_{i}$ as element.

When there are only a few large coefficients in the sparse vector α, the signal ***x*** can be expressed as sparsely represented under the basis Ψ. The elements $\alpha_{i}$ are sorted by a certain order to exhibit an exponential decay trend, and only *K* (K≪N) elements are large coefficients, that we can refer to signal ***x*** as K-sparse.

**Figure 4:**
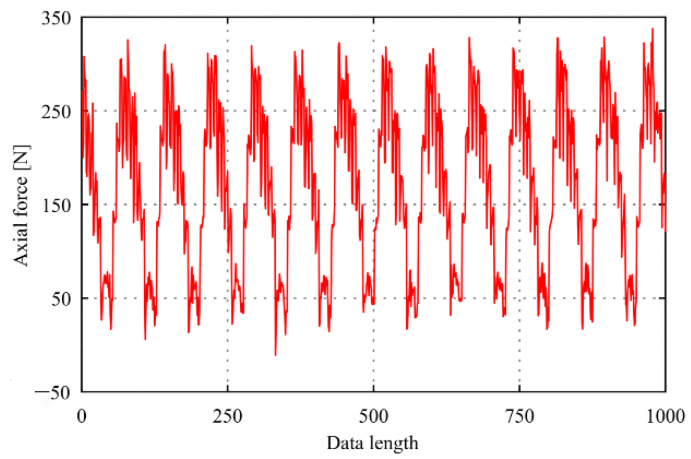
Waveform of original signal.

In this paper, the cutting force test signal is collected at a 500 Hz sampling frequency in the self-designed cutting experiment by the intelligent tool holder and selects two seconds of data, as shown in Figure 4. It can be observed that there are few points in the original cutting force signal with a value of zero or approximately zero. Thus, the cutting force signal is non-sparse in the time domain and needs to find a transform domain to sparse representation.

As the basis of signal analysis theory, Fourier Transform (FT) constructs the signals relationship between time domain and frequency domain. However, the cutting force signal obtained by the intelligent tool holder system is a continuous analog signal and cannot be recognized by the computing device directly, thus it should be converted to a discrete digital signal to process. Discrete Fourier Transform (DFT) is an FT method that can realize the spectrum analysis of discrete signals with a finite length in both the time domain and frequency domain. Its form is as follows in Equation (2), and is rewritten into matrix form as follows in Equation (3):

(2) $$x\left(n\right)=\frac{1}{N}\sum_{k=0}^{N-1}X\left(k\right)\cdot \exp\left(j\frac{2\pi }{N}nk\right)=\frac{1}{N}\sum_{k=0}^{N-1}X\left(k\right)\cdot W_{N}^{-nk}$$

(3) $$\left[\begin{array}{c} x_{0}\\ x_{1}\\ \vdots \\ x_{N-1} \end{array}\right]=\left[\begin{array}{cccc} \frac{1}{N} & \frac{1}{N} & \ldots & \frac{1}{N}\\ \frac{1}{N} & \frac{1}{N}\cdot W_{N}^{-1} & \cdots & \frac{1}{N}\cdot W_{N}^{-\left(N-1\right)}\\ \vdots & \vdots & \ddots & \vdots \\ \frac{1}{N} & \frac{1}{N}\cdot W_{N}^{-\left(N-1\right)} & \ldots & \frac{1}{N}\cdot W_{N}^{-{\left(N-1\right)}^{2}} \end{array}\right]\cdot \left[\begin{array}{c} X_{0}\\ X_{1}\\ \vdots \\ X_{N-1} \end{array}\right]$$

where x(n) is the finite-length sequence in time domain, n=0,1,…,N−1, X(k) is the finite-length sequence in frequency domain, k=0,1,…,N−1, *j* denotes the imaginary number and $W=\exp\left(-j\frac{2\pi }{N}\right)$.

Which is

(4) $$x=\Psi^{T}\cdot X$$

**Figure 5:**
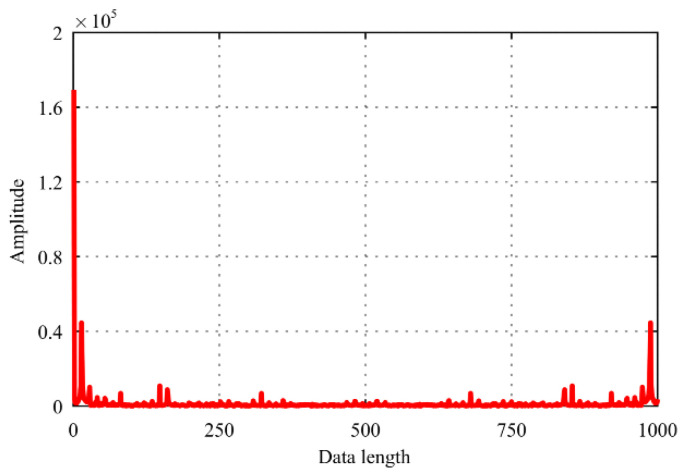
Waveform of transformed signal.

The data as shown in Figure 4. is transformed by DFT to obtain a waveform diagram in the frequency domain, as shown in Figure 5. It can be seen that the cutting force signal exhibits an exponential decay trend, and the large coefficients are few enough to refer to it as sparse in the frequency domain. The maximum coefficient value is defined as $cof_{\max}$, and the number of coefficient values more than 0.01 times $cof_{\max}$ is 103. Therefore, the cutting force signal can be sparsely expressed after DFT transformation, and the sparsity *K* = 103.

#### 3.2.2. Construction of Sampling Signals

The sparse signal needs to be mapped to a low-dimensional space, as follows in Equation (5), and the compression ratio is defined as c=(N−M)/N. Moreover, the sampling matrix should satisfy the restricted isometry property (RIP), which means that the measuring matrix is not related to the sparse matrix [20].

(5) $$y=\Phi \cdot x=\Phi \cdot \Psi \cdot \alpha =\tilde{\Phi }\cdot \alpha$$

where, *y* is the compressed signal, *M* × 1 and *M* is the measuring value, Φ is the measuring matrix, *M* × *N* and M≪N, $\tilde{\Phi }$ is the sampling matrix.

In this paper, a Gaussian random measuring matrix is used to construct the sampling matrix. When the length of the original signal ***x*** is *N* and the sparsity is *K*, the measuring value *M* only needs to satisfy the relationship M≥c·K·log(N/K) to meet RIP with a very high probability. Therefore, when the measuring value *M* is no less than 235, the high-precision reconstruction of the cutting force signal shown in Figure 4 can be realized with a maximum compression ratio of 76.5%.

In order to further improve the compression performance of the measurement matrix and the accuracy of signal reconstruction, a measuring matrix optimization method combining approximate (orthogonal upper triangle, QR) decomposition and minimum average mutual coherence coefficient is proposed.

According to the matrix decomposition theory, the correlation of the matrix decreases with the increase of the minimum singular value [21]. As a special matrix maximum rank decomposition method, the standard QR decomposition can decompose Φ into a square matrix ***Q*** and an upper triangular matrix ***R*** for any $\Phi \in {\mathbb{C}}_{r}^{M\times N}$, as follows in Equation (6). Besides, matrix ***Q*** satisfies relationship $Q^{H}\cdot Q=I_{r}$, where $I_{r}$ denotes the r-order identity matrix.

(6) $$\Phi ={\left(Q\cdot R\right)}^{T}$$

In approximate QR decomposition, the diagonal matrix $\bar{R}$ is obtained by keeping the elements on the main diagonal of ***R*** unchanged and setting other position elements to zero. Subsequently, ***Q*** and $\bar{R}$ are combined to construct a new measuring matrix $\bar{\Phi }$. Therefore, the Gaussian random measuring matrix is optimized by the approximate QR decomposition with the minimum singular value larger than the original matrix, which is proved as follows in Equation (7).

(7) $$\begin{array}{ll} \sigma_{\min}\left(\Phi \right) & =\sqrt{\lambda_{\min}\cdot \left(\Phi \cdot \Phi^{T}\right)}=\sqrt{\underset{v}{\min}\frac{v^{T}\cdot \Phi \cdot \Phi^{T}\cdot v}{v^{T}\cdot v}}=\sqrt{\underset{v}{\min}\frac{v^{T}\cdot R\cdot R^{T}\cdot v}{v^{T}\cdot v}}\\ & \leq \sqrt{\frac{{\bar{v}}^{T}\cdot R\cdot R^{T}\cdot \bar{v}}{v^{T}\cdot v}}=\sqrt{\lambda_{\min}\cdot \left(\bar{R}\cdot {\bar{R}}^{T}\right)}=\sqrt{\lambda_{\min}\cdot \left(\bar{\Phi }\cdot {\bar{\Phi }}^{T}\right)}=\sigma_{\min}\left(\bar{\Phi }\right) \end{array}$$

where, $\sigma_{\min}$ is the minimum singular value of the matrix, $\lambda_{\min}$ is the minimum eigenvalue of the matrix, ***v*** and $\bar{v}$ are column vectors, which corresponding to the minimum elements in the diagonal of matrix ***R*** and $\bar{R}$ are taken as 1 and the others are taken as 0 respectively.

In order to further reduce the correlation of matrix, the minimum average mutual coherence coefficient method is introduced, which calculates the t-average mutual coherence coefficient defined in Equation (8), as proposed by Elad [22].

(8) $$\mu_{t}\left(\tilde{\Phi }\right)=\frac{\sum_{1\leq i,j\leq N,i\neq j}\max\left(\left|g_{ij}\right|\geq t\right)\cdot \left|g_{ij}\right|}{\sum_{1\leq i,j\leq N,i\neq j}\max\left(\left|g_{ij}\right|\geq t\right)}$$

where $g_{ij}$ is the element in the Gram matrix ***G*** and $G={\tilde{\Phi }}^{T}\cdot \tilde{\Phi }$, ***t*** is a non-negative parameter.

The maximum correlation coefficient of the standard Gaussian random measuring matrix, the Gaussian random measuring matrix optimized by the approximate QR decomposition, the Gaussian random measuring matrix optimized by the minimum average mutual coherence coefficient and the Gaussian random measuring matrix optimized by the proposed algorithm are 0.7463, 0.5673, 0.5999, and 0.5185, respectively. Therefore, the algorithm proposed in this paper is effective in reducing the correlation of the measuring matrix. Namely, the sampling matrix constructed by the optimized Gaussian random measuring matrix has a higher probability of meeting the RIP necessary to realize high-precision recovery of signal.

#### 3.2.3. Recovery of Sampling Signals

The sampling signal ***y*** cannot be directly observed by the user when transmitted to the upper computer, due to it not being a time domain signal and having a size of *M ×* 1 not *N ×* 1. Therefore, the *M ×* 1 sampling signal ***y*** needs to be reconstructed to *N ×* 1 recovery signal $\tilde{x}$. Owing to M≪N, there is an infinite solution for solving the Equation (5) when the sampling signal ***y*** is reconstructed to recovered signal $\tilde{x}$, which is an NP-Hard problem. However, there is an optimal solution to realize signal reconstruction by adding appropriate constraints to render the recovered signal $\tilde{x}$ as sparse as possible, as follows in Equation (9).

(9) $$\min{\left\|\alpha \right\|}_{0}\;\;\;s.t.\;y=\tilde{\Phi }\cdot \alpha$$

Therefore, the signal reconstruction problem is transformed into the optimization problem, which can be solved with low computational complexity and high efficiency reconstruction by using the greedy algorithm. As one of the classic greedy algorithms, an Orthogonal Matching Pursuit (OMP) algorithm [23] could pick a non-zero element in the sparse vector α based on the best matching principle in iteration at first. Then, the pseudo-inverse transform is utilized to correct the selected non-zero element values until all non-zero elements in the sparse vector α are selected. Finally, the sparse basis Ψ is multiplied with the sparse vector α to obtain the recovered signal $\tilde{x}$. The algorithm updates the atomic library in each iteration only by picking up one element that best matches the residual, which may lead to a longer reconstruction time. By introducing backtracking theory into the OMP algorithm, a new algorithm is formed called the Compressive Sampling Matching Pursuit (CoSaMP) algorithm that selects multiple more related atoms from the atomic library and culls some of the atoms in iteration [24]. The reconstruction efficiency is greatly improved by the CoSaMP algorithm.

**Figure 6:**
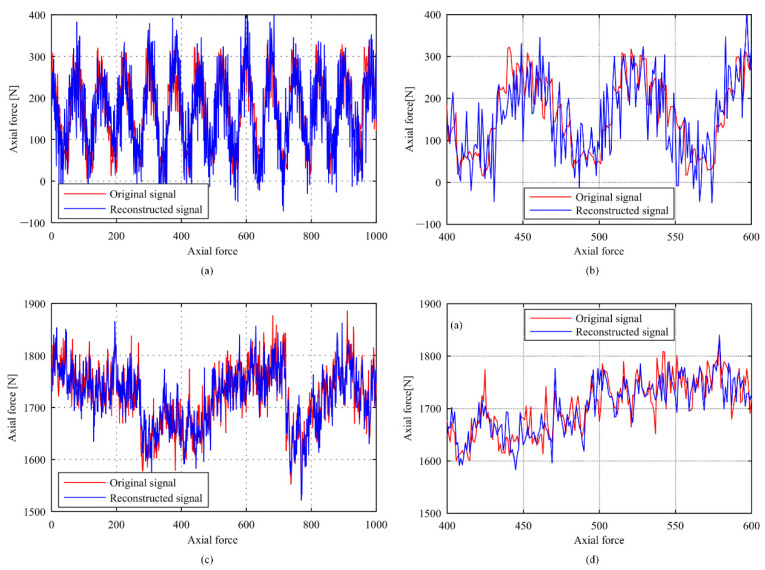
Reconstruction effect comparison of (**a**) milling force, (**b**) local enlarged view of milling force, (**c**) drilling force and (**d**) local enlarged view of drilling force.

When the DFT basis is used to sparsely represent the cutting force signal and the optimized Gaussian random measuring matrix is used to construct sampling signal, the reconstruction effect of the force signal during the drilling process by CoSaMP algorithm is shown in Figure 6. When the compression ratios *c* = 0.7, the reconstruction error is 2.10% and the reconstruction time is 0.016 s for milling cutting force signal as shown in Figure 4. When the compression ratios *c* = 0.6, the reconstruction error is 1.10% and the reconstruction time is 0.052 s for drilling cutting force signal with the sparsity *K* = 201.

## 4. Scheme of Variable Parameter Peck Drilling Experiments

### 4.1. Scheme of Cutting Experiments for Intelligent Tool Holder System

**Figure 7:**
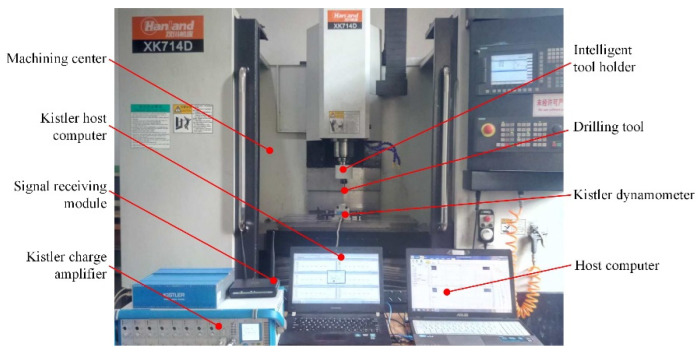
Photograph of the cutting experiment.

To verify the performance of the cutting force measurement in a real-time machining process, several cutting tests were carried out. For the actual cutting process, there are many factors affecting the cutting force, thus it is hard to calculate the cutting force precisely through the theoretical formulas. The actual measuring effect can only be assessed by comparing the measurement results of the high-precision reference dynamometer. In this paper, a Kistler 9119AA2 plate dynamometer was selected as the reference dynamometer, a 10 mm straight handle twist drill made of HSS with helix angle of 30 and apex angle of 135 (Shanghai Hashen Tools Co., Ltd., Shanghai, China) was used and assembled in the intelligent tool holder. The experiments were conducted by drilling 45 steel under dry cutting conditions using a XK714D three-axis vertical machining center (Hanchuan CNC Machine Tools Co., Ltd., Hanzhong, China). The cutting experiment platform was built as shown in Figure 7.

### 4.2. Scheme of Drilling Experiments for Ti-CFRP-Ti Laminated Stacks

The laminated workpiece consists of two titanium alloy layers (TC4) and one CFRP layer (T300) with inner filaments laid in directions of 45°, −45°, 0°, 90° with the size of 60 × 40 × 5 mm^3^ and 60 × 40 × 6 mm^3^, respectively. The properties of these two materials are shown in Table 1.

**Figure 8:**
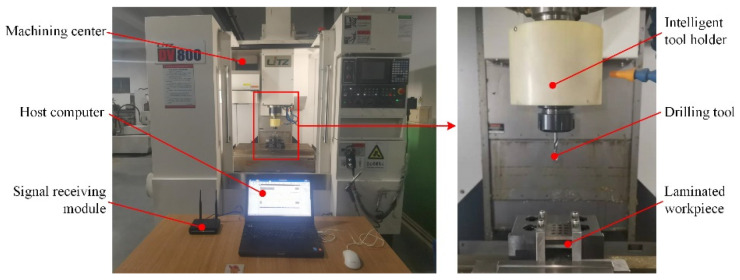
Photographs of the drilling experiment for Ti-CFRP-Ti laminated stacks.

The experiment was carried out on a DV800 three-axis vertical machining center. The intelligent tool holder was installed on the spindle of the machine tool. Based on the specially designed fixture, titanium alloy and CFRP were stacked layer by layer to form a laminated workpiece where the number of layers can be adjusted randomly. Moreover, the titanium alloy and CFRP were cut along the axial direction after drilling, and the Form Talysurf i60 desktop roughness profiler was utilized to measure the surface roughness of the hole wall. The experimental platform is shown in Figure 8.

A cemented carbide twist drill was selected as the cutting tool according to the previous research results [25] with a 6 mm diameter, 115° top angle, 25° helix angle, 10° rear angle of outer edge, and 12° inclination angle of transverse edge.

There are six drilling parameters in the process of variable parameter peck drilling: spindle speed of upper titanium alloy layer $n_{1}$, feed rate of upper titanium alloy layer $f_{1}$, spindle speed of CFRP layer $n_{2}$, feed rate of CFRP layer $f_{2}$, spindle speed of lower titanium alloy layer $n_{3}$ and feed rate of lower titanium alloy layer $f_{3}$. According to the actual production and processing conditions, six factors and five levels of orthogonal experiments were designed for the above drilling parameters. Table 2 shows the factors and levels of orthogonal experiments, and an average value of four repeated drilling holes in each group was calculated as the final measurement result.

## 5. Experimental Results and Analysis

### 5.1. Cutting Experimental Results of Intelligent Tool Holder System

**Figure 9:**
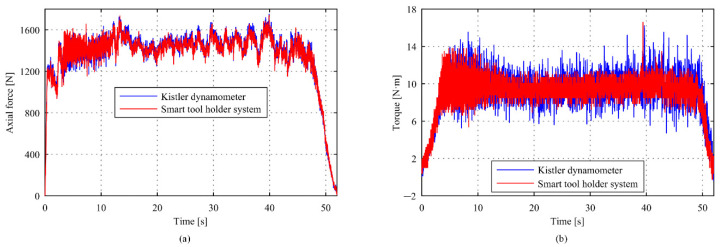
Comparison of the intelligent tool holder system with Kistler dynamometer on the (**a**) axial force and (**b**) torque (*n* = 500 r/min, *v*_f_ = 50 mm/min).

**Figure 10:**
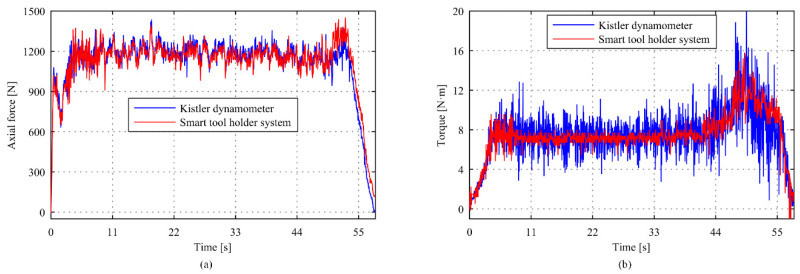
Comparison of the intelligent tool holder system with Kistler dynamometer on the (**a**) axial force and (**b**) torque (*n* = 600 r/min, *v*_f_ = 50 mm/min).

The comparisons of torque and axial force measurement results between the intelligent tool holder and reference dynamometer at a spindle speed *n* = 500 r/min and feed speed *v*_f_ = 50 mm/min are shown in Figure 9. The mean value of the tool holder system and reference dynamometer measurement in the axial force direction was 1355.7 N and 1365.4 N with a deviation of 0.7%, and that in the torque direction was 9.015 N·m and 9.1735 N·m with a deviation of 1.73%. Figure 10 shows the measurement results at a spindle speed *n* = 500 r/min and feed speed *v*_f_ = 50 mm/min. It can be seen that the mean value of the tool holder system and reference dynamometer measurement in the axial force direction was 1113.0 N and 1114.3 N with a deviation of 0.12%, and that in the torque direction was 7.9064 N·m and 7.4122 N·m with a deviation of 1.02%. A difference between the measurement method and sensing position will lead to the deviation of the measurement results between the intelligent tool holder system and the dynamometer. Overall, the deviation of torque is slightly larger than the axial force. The reason is that the semiconductor sensor adopted for the measurement of axial force comes with a higher sensitivity and resolution. Considering the bonding process, the resistance sensor is used for torque measurement with slightly lower sensitivity. Generally, the maximum deviations do not exceed 2%, which can meet the actual machining requirements.

The results of the drilling test show that the intelligent tool holder system and reference dynamometer bear good consistency in measuring performance, which means that the system has high measuring accuracy and can be used to measure cutting force in actual production and processing.

### 5.2. Drilling Experimental Results of Ti-CFRP-Ti Laminated Stacks

The orthogonal experiments of drilling parameters were carried out by changing the parameters at the interface region of the two materials. The main effect plot was obtained by range analysis, so as to analyze the axial force and surface roughness of each layer. The mathematical regression of axial force and surface roughness was carried out by statistical method.

#### 5.2.1. Analysis of Axial Force

**Figure 11:**
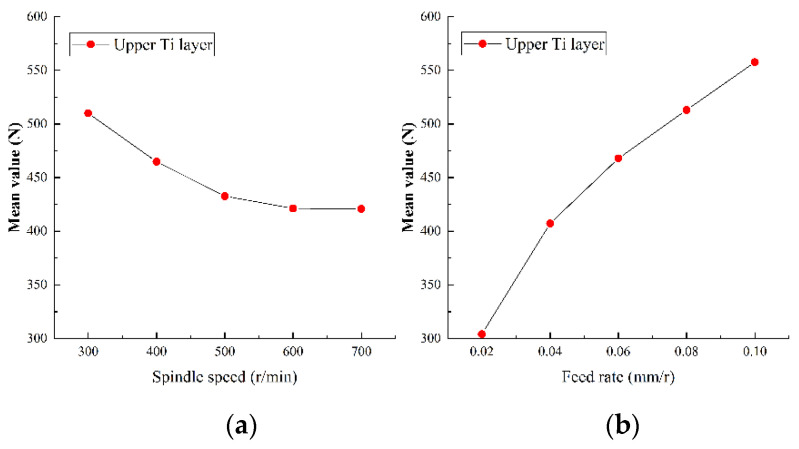
Main effect plots of axial force for drilling upper titanium alloy in (**a**) spindle speed and (**b**) feed rate.

**Figure 12:**
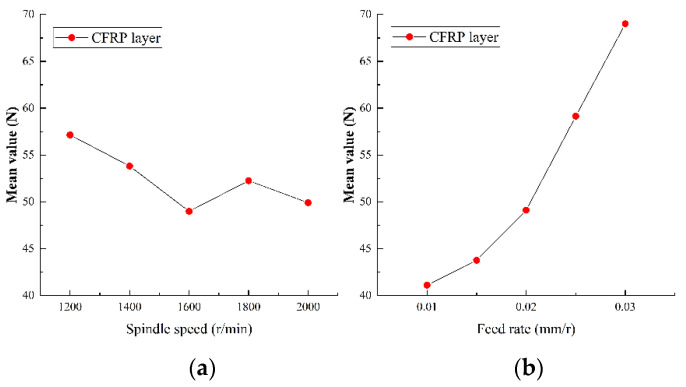
Main effect plots of axial force for drilling CFRP in (**a**) spindle speed and (**b**) feed rate.

**Figure 13:**
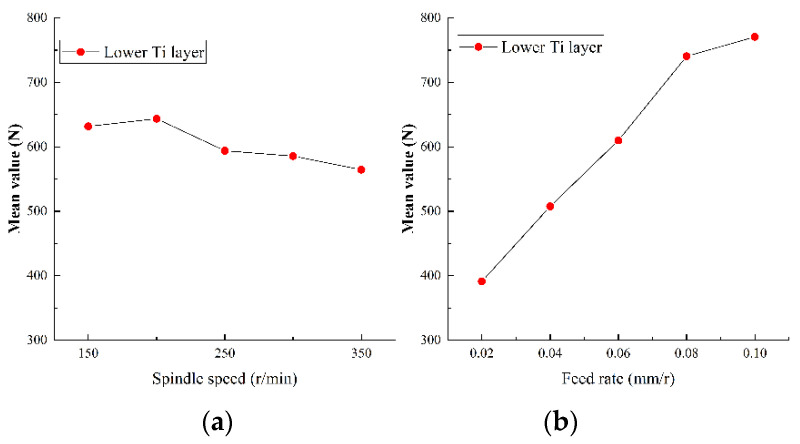
Main effect plots of axial force for drilling lower titanium alloy in (**a**) spindle speed and (**b**) feed rate.

As the most important physical quantity in drilling process, the axial force has an important impact on tool wear and hole quality. The range analysis of the axial force in the experiment results of Ti-CFRP-Ti peck drilling with variable parameters was carried out to study the influence of drilling parameters on the axial force. Figure 11, Figure 12 and Figure 13 show the main effect plots of axial force for drilling upper titanium alloy, CFRP and lower titanium alloy.

It can be observed that the variation trend of axial force of the titanium alloy and CFRP with drilling parameters is essentially the same, but the specific values differ. On one hand, the axial force fluctuates with an increase of spindle speed, but tends to decrease as a whole. The reason may be that an increase of cutting speed changes the interface friction coefficient, while the strength and hardness of machining materials become reduced due to heat accumulation, thus reducing the cutting load. On the other hand, the axial force increases linearly with feed rate, as the increase of feed rate makes the drilling area larger, and the friction in drilling deformation area and the resistance of drilling material will lead to an increase of the axial force. In addition, the variation range of the axial force caused by the spindle speed is relatively small, but the variation of the axial force caused by the feed rate is relatively large during the whole drilling process of Ti-CFRP-Ti laminated stacks. Therefore, the influence of feed rate on the axial force in Ti-CFRP-Ti peck drilling process is much greater than that of spindle speed.

The mathematical model of the axial force and drilling parameters was established by regression analysis of the data of the axial force in the peck drilling of Ti-CFRP-Ti laminated stacks with variable parameters in order to further study and predict the relationship between the axial force and drilling parameters. The exponential relation was used as a regression model of the axial force, as follows in Equation (10).

(10) $$F_{z}=C\cdot n^{K_{n}}\cdot f^{K_{f}}$$

where C is the regression coefficient, n and f are the spindle speed and feed rate used for drilling each layer, $K_{n}$ and $K_{f}$ are the regression indexes of n and f. The axial force data of each layer collected in the variable parameter peck drilling of Ti-CFRP-Ti laminated stacks were substituted into the formulas, respectively, and the mathematical models of the axial force and drilling parameters of different layers were obtained.

For drilling of upper titanium alloy:

(11) $$F_{z1}=10^{3.732}\cdot n^{-0.2273}\cdot f^{0.3754}$$

For drilling of CFRP:

(12) $$F_{z2}=10^{3.342}\cdot n^{-0.230}f^{0.4588}$$

For drilling of lower titanium alloy:

(13) $$F_{z3}=10^{3.737}\cdot n^{-0.1744}\cdot f^{0.4352}$$

Goodness of fit is generally used to measure the fitting degree of the regression model to experimental value to verify the accuracy of the regression model. The statistical measure of goodness of fit is the coefficient of solution (determinate coefficient), which is expressed by $R^{2}$. As follows in Equation (14).

(14) $$R^{2}=\frac{\sum {\left(\hat{y}-\bar{y}\right)}^{2}}{\sum {\left(y-\bar{y}\right)}^{2}}=1-\frac{\sum {\left(y-\hat{y}\right)}^{2}}{\sum {\left(y-\bar{y}\right)}^{2}}$$

where y denotes the experimental value of the regression index to be returned, $\bar{y}$ denotes the average value of all the experimental values, and $\hat{y}$ denotes the regression value of the regression index to be returned.

It is believed that the regression model has high reliability and can accurately predict the test values when $R^{2}\geq 0.8$. The coefficients of the regression model for the axial force of each layer are $R_{1}{}^{2}=0.8896$, $R_{2}{}^{2}=0.8063$ and $R_{3}{}^{2}=0.8892$, thus the mathematical model of axial force is shown to be quite accurate.

#### 5.2.2. Analysis of Surface Roughness of Hole Wall

**Figure 14:**
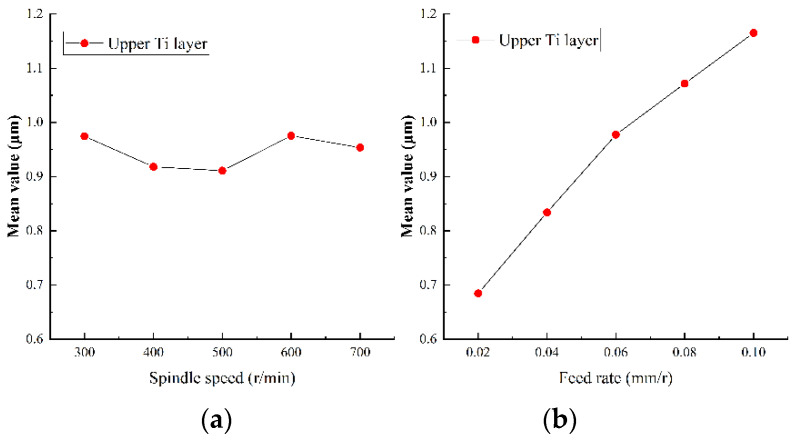
Main effect plots of surface roughness of hole wall for drilling upper titanium alloy in (**a**) spindle speed and (**b**) feed rate.

**Figure 15:**
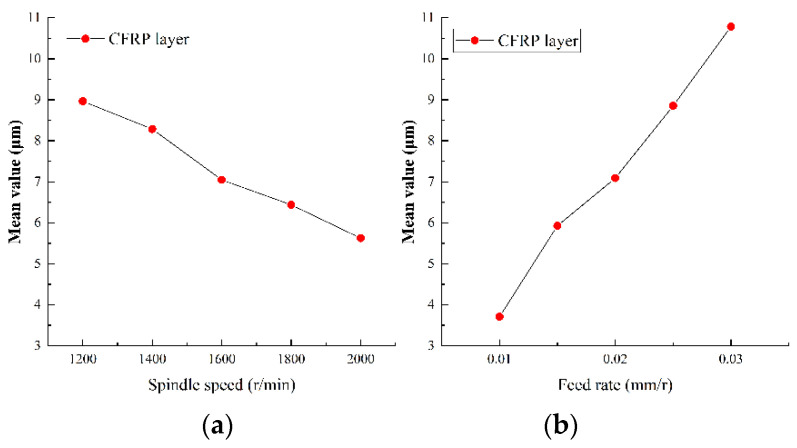
Main effect plots of surface roughness of hole wall for drilling CFRP in (**a**) spindle speed and (**b**) feed rate.

**Figure 16:**
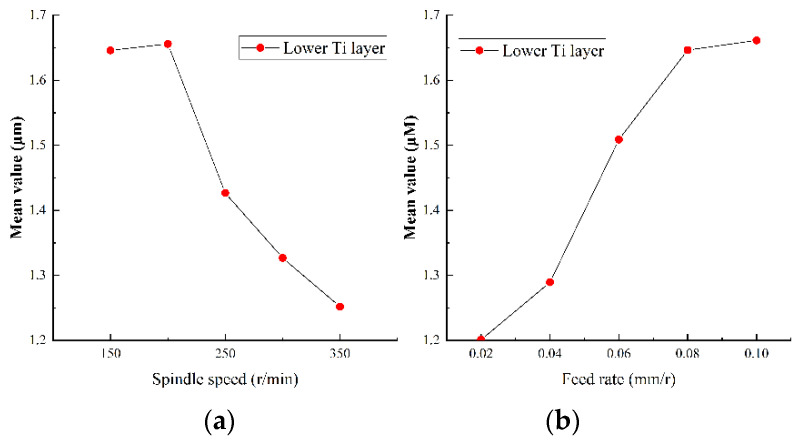
Main effect plots of surface roughness of hole wall for drilling lower titanium alloy in (**a**) spindle speed and (**b**) feed rate.

Surface roughness of the hole wall is an important index to measure drilling quality. Small surface roughness can not only bring reliable assembly accuracy, but also improves the reliability and service life of parts. The surface roughness of Ti-CFRP-Ti laminated stacks was measured after variable parameter peck drilling and the results were obtained by main effect analysis, as shown in Figure 14, Figure 15 and Figure 16.

It can be observed that the variation trend of the hole wall surface roughness with drilling parameters is similar to that of cutting force. On one hand, the hole wall surface roughness of CFRP decreases with an increase of spindle speed, while the hole wall surface roughness of titanium alloy shows a local fluctuation with spindle speed, however, it also shows a negative correlation. The reason may be that lower spindle speed is more likely to produce built-up edges and scales, which will scrape the machined surface. On the other hand, the hole wall surface roughness of each layer shows a nearly linear growth trend with the feed rate, which is due to the increase of chips which strengthen the scribing and friction effects on the surface of the hole wall. In addition, it was found that the surface roughness of CFRP layer was significantly greater than that of titanium alloy layer, The reason may be that CFRP is made of high-strength carbon fiber with a rough section, and the chip removal process of the lower titanium alloy layer will scratch the hole wall of CFRP layer.

Regression analysis was used to analyze the experimental results and a function model of the hole wall surface roughness on drilling parameters was established in order to obtain the relationship between the hole wall roughness and drilling parameters. The researches in references [1,3] show that there is a quadratic polynomial relationship between surface roughness and drilling parameters. Therefore, the function model can be expressed as follows in Equation (15).

(15) $$R=A\cdot n^{2}+B\cdot f^{2}+C\cdot n\cdot f+D\cdot n+E\cdot f+F$$

where *A*, *B*, *C*, *D*, *E*, *F* are regression constants, *n* and *f* are spindle speed and feed rate, respectively.

The surface roughness of each hole wall was measured separately, and a mathematical model of the hole wall surface roughness and drilling parameters of Ti-CFRP-Ti laminated stacks was obtained.

For drilling of upper titanium alloy layer:

(16) $$R_{a1}=0.000001n^{2}-28.9f^{2}+0.00158\cdot n\cdot f-0.00108n+8.67f+0.776$$

For drilling of CFRP layer:

(17) $$R_{a2}=0.000001n^{2}+69f^{2}+0.156\cdot n\cdot f-0.0094n+89f+13.9$$

For drilling of lower titanium alloy layer:

(18) $$R_{a3}=-0.000001n^{2}-40.9f^{2}-0.0636\cdot n\cdot f+0.00216n+27.19f+0.502$$

The coefficients of the regression model for the roughness of each layer were $R_{1}{}^{2}=0.8480$, $R_{2}{}^{2}=0.8099$ and $R_{3}{}^{2}=0.8489$. It can be observed that the surface roughness mathematical model of variable parameter peck drilling of Ti-CFRP-Ti laminated stacks established in this paper is quite accurate.

### 5.3. Multi-Objective Optimization of Drilling Parameters Based on Analytic Hierarchy Process (AHP)

The multi-objective optimization model includes three elements: design variables, objective functions and constraints. Surface roughness, axial force and material removal rate are taken as objective functions of the optimization problem. Spindle speed and feed rate are taken as design variables of optimization problem in order to investigate the influence of spindle speed and feed rate on the objective function. In addition, the constraints refer to the range of drilling parameters. The multi-objective optimization models can be listed according to the actual situations as follows in Equation (19).

(19) $$\left\{ \begin{array}{l} \min Y(n_{i},f_{i})=(Ra_{i}(n_{i},f_{i}),F_{zi}(n_{i},f_{i}),P_{i}(n_{i},f_{i}))\\ s.t.\left\{ \begin{array}{l} n_{i(\min)}\leq n_{i}\leq n_{i(\max)}\\ f_{i(\min)}\leq f_{i}\leq f_{i(\max)} \end{array}\right. \end{array}\right.$$

where *P_i_*, $n_{i}$ and $f_{i}$ are the material removal rate, spindle speed and feed rate when drilling the layer i(i=1,2,3).

On one hand, the objectives are often mutually constrained, and generally unable to achieve several optimal objective functions at the same time in multi-objective optimization. The improvement of one objective often leads to the reduction of other objectives, so only coordination and compromise between them can be carried out to optimize each objective as far as possible. Compared with the single objective optimization problem, the solution of the multi-objective optimization problem is not unique, but a group of optimal solutions verified by experiments. On the other hand, the regularity between the variable parameters selected by each layer and the target parameters obtained is relatively independent.

Firstly, a unified evaluation function for the optimization of each layer was established by transforming multi-objective optimization into single-objective optimization based on compromise programming approach. Then, the weight factor of each layer material objective function was determined by AHP, so as to construct the evaluation function. Finally, the genetic algorithm was used to optimize the parameters of the evaluation function, and the unique solution was obtained.

#### 5.3.1. Establishment of Multi-Objective Optimization Model

It is often desirable that the drilling efficiency can be improved on the premise of guaranteeing the processing quality. Therefore, an optimization model with the objective function of the hole surface roughness, axial force and material removal rate was established. The function of the axial force and the surface roughness of the hole wall is the mathematical model obtained by regression analysis in the previous paper. The material removal rate function is as follows in Equation (20).

(20) $$Q=\frac{\pi \cdot n\cdot f\cdot d^{2}}{4}$$

The drill diameter *d* = 6 mm is a fixed value and the minimum value of the function is required in the optimization process, thus the negative number of material removal rate is taken as the objective function, which is shown in the Equation (21).

(21) $$P=-4Q=\pi \cdot n\cdot f\cdot d^{2}$$

The linear weighting method is generally used to transform a multi-objective into a single-objective, but the evaluation function constructed is only applicable to the problem assuming that each objective function is convex. Moreover, the order of magnitude of the objective function in this paper is quite different. Therefore, the compromise programming approach was selected to keep each objective function in the same order of magnitude. The mathematical expression of the compromise programming approach is as follows in Equation (22).

(22) $$F={\left\{\sum_{k=1}^{m}w_{k}{}^{2}{\left[\frac{f_{k}-f_{k}^{\min}}{f_{k}^{\max}-f_{k}^{\min}}\right]}^{2}\right\}}^{\frac{1}{2}}$$

where $w_{k}$ is the weight factor of the objective function, which is obtained by AHP. $f_{k}$ is the corresponding objective function, $f_{k}^{\max}$ is the maximum of the objective function and $f_{k}^{\min}$ is the minimum of the objective function.

**Figure 17:**
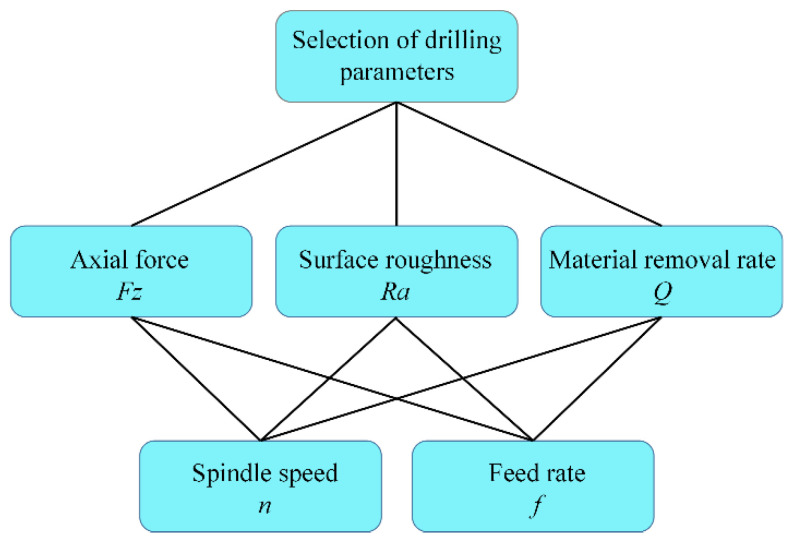
Optimizing scheme.

The optimization is divided into a decision layer (optimal scheme), criterion layer (surface roughness of hole wall, axial force and material removal rate) and scheme layer (spindle speed and feed rate), as shown in Figure 17. Multi-objective functions include surface roughness, axial force and material removal rate. Three unknown weight factors need to be determined to construct evaluation function by using compromise programming approach for each layer. These weight factors were calculated by AHP. The subjective judgment was ranked quantitatively on the basis of a hierarchical structure model according to the analytic steps and measurement theory of AHP. In other words, the criterion layer objectives of each layer were compared to determine the judgment matrices and to calculate the comprehensive weights of each element according to the actual situation. Finally, the rationality of the weight factors were verified by calculating the consistency ratio, thus the weight factors of $F_{z}$, $R_{a}$ and Q were $\omega_{1}$, $\omega_{2}$ and $\omega_{3}$.

The relative importance of three objects is determined to construct the mutual judgment matrix according to the scale table [26]. The judgment matrix W is constructed as follows in Equation (23).

(23) $$W=\left[\begin{array}{ccccc} w_{11} & \cdots & w_{1i} & \cdots & w_{1n}\\ \vdots & \vdots & \vdots & \vdots & \vdots \\ w_{j1} & \cdots & w_{ji} & \cdots & w_{jn}\\ \vdots & \vdots & \vdots & \vdots & \vdots \\ w_{n1} & \cdots & w_{ni} & \cdots & w_{nn} \end{array}\right]$$

where n is the number of weight factors, $w_{i}$ and $w_{j}(i=1,2,\cdots ,6)$ are the weight factors, $w_{ji}=w_{j}/w_{i}$ is the relative importance of $w_{j}$ to $w_{i}$.

For the optimization model of drilling parameters of the upper titanium alloy, it was found that the axial force in drilling the titanium alloy layer is large and the processing system vibrated easily. However, the surface roughness of the hole wall is almost the same value needed to meet accuracy requirements. Therefore, the importance of the upper titanium alloy is $\omega_{1}>\omega_{2}>\omega_{3}$, and the judgment matrix is given according to the scale table as follows in Equation (24).

(24) $$W_{1}=\left[\begin{array}{ccc} 1 & 5 & 3\\ 1/5 & 1 & 1/3\\ 1/3 & 3 & 1 \end{array}\right]$$

There are several methods to calculate the ranking weight of each index from the judgment matrix. The most widely used method is the eigenvalue method (EM). It is used to calculate the weight of each index as follows.

Let $\omega ={\left(\omega_{1},\omega_{2},\omega_{3}\right)}^{T}$ be the ranking weight vector of the judgment matrix W. Then the following equation is constructed.

(25) Wω=λω

where λ is the eigenvalue of the judgment matrix, and its maximum value $\lambda_{\max}$ exists and is unique, ω is the corresponding eigenvector and can be used as ranking weight vector after normalization. Therefore, $\omega ={\left(0.6307,0.1047,0.2583\right)}^{T}$ is obtained.

The consistency test and random consistency test of the judgment matrix are carried out in order to ensure the accuracy and reliability of the judgment matrix and avoid the influence of subjective factors on the judgment matrix. The weight factors pass the test by calculation.

It was observed that the surface roughness of CFRP layer was large, the quality of the drilling was poor, and the axial force was stable. There should be $\omega_{2}>\omega_{3}>\omega_{1}$ for CFRP. The weight vector ω=(0.0936,0.6267,0.2797) of the objective function in the optimization model of drilling parameters for CFRP layers can be obtained with the above method.

It was found that the value of the axial force was large and the fluctuation was large when drilling lower titanium alloy. In addition, the spindle speed was low considering drilling temperature and other factors, which lead to a low processing efficiency. The surface roughness of the hole wall of lower titanium alloy was larger compared with the upper titanium alloy, but essentially satisfies the manufacturing requirements. Thus, there is $\omega_{1}>\omega_{2}>\omega_{3}$ in the importance of the objective function in the lower titanium alloy layer. The weight vector $\omega ={\left(0.6483,0.2297,0.122\right)}^{T}$ of the objective function in the optimization model of drilling parameters for lower titanium alloy layer can be obtained with the above method.

The optimization functions for each layer are established as follows.

(26) $$\left\{ \begin{array}{l} \min Y_{1}={\left(0.6307\right)}^{2}\frac{{\left(F_{z1}(n_{1},f_{1})-F_{z1}^{\min}\right)}^{2}}{\left(F_{z1}^{\max}-F_{z1}^{\min}\right)}+{\left(0.1047\right)}^{2}\frac{{\left(R_{a1}(n_{1},f_{1})-R_{a1}^{\min}\right)}^{2}}{\left(R_{a1}^{\max}-R_{a1}^{\min}\right)}-{\left(0.2583\right)}^{2}\frac{{\left(Q(n_{1},f_{1})-Q^{\min}\right)}^{2}}{\left(Q^{\max}-Q^{\min}\right)}\\ s.t.\left\{ \begin{array}{l} 300r/\min\leq n_{1}\leq 700r/\min\\ 0.02mm/r\leq f_{1}\leq 0.1mm/r \end{array}\right. \end{array}\right.$$

(27) $$\left\{ \begin{array}{l} \min Y_{2}={\left(0.0936\right)}^{2}\frac{{\left(F_{z2}(n_{2},f_{2})-F_{z2}^{\min}\right)}^{2}}{\left(F_{z2}^{\max}-F_{z2}^{\min}\right)}+{\left(0.6267\right)}^{2}\frac{{\left(R_{a2}(n_{2},f_{2})-R_{a2}^{\min}\right)}^{2}}{\left(R_{a2}^{\max}-R_{a2}^{\min}\right)}-{\left(0.2797\right)}^{2}\frac{{\left(Q(n_{2},f_{2})-Q^{\min}\right)}^{2}}{\left(Q^{\max}-Q^{\min}\right)}\\ s.t.\left\{ \begin{array}{l} 1200r/\min\leq n_{2}\leq 2000r/\min\\ 0.01mm/r\leq f_{2}\leq 0.3mm/r \end{array}\right. \end{array}\right.$$

(28) $$\left\{ \begin{array}{l} \min Y_{3}={\left(0.6483\right)}^{2}\frac{{\left(F_{z3}(n_{3},f_{3})-F_{z3}^{\min}\right)}^{2}}{\left(F_{z3}^{\max}-F_{z3}^{\min}\right)}+{\left(0.2297\right)}^{2}\frac{{\left(R_{a3}(n_{3},f_{3})-R_{a3}^{\min}\right)}^{2}}{\left(R_{a3}^{\max}-R_{a3}^{\min}\right)}-{\left(0.122\right)}^{2}\frac{{\left(Q(n_{3},f_{3})-Q^{\min}\right)}^{2}}{\left(Q^{\max}-Q^{\min}\right)}\\ s.t.\left\{ \begin{array}{l} 150r/\min\leq n_{1}\leq 300r/\min\\ 0.02mm/r\leq f_{1}\leq 0.1mm/r \end{array}\right. \end{array}\right.$$

where $Y_{i}$ is the evaluation function, $F_{zi}$ is the regression equation of force, $R_{ai}$ is the regression equation of roughness, Q is the material removal rate, $F_{zi}^{\min}$, $F_{zi}^{\max}$, $R_{ai}^{\min}$, $R_{ai}^{\max}$, $Q^{\min}$ and $Q^{\max}$ are the extreme values under the corresponding constraints when drilling each layer.

#### 5.3.2. Solution Based on Genetic Algorithms

Compared with traditional optimization methods, genetic algorithm has the advantages of good convergence, strong global search ability, high robustness and scalability, etc. The genetic algorithm in MATLAB was used to solve the above optimization model. Setting population size, crossover probability, crossover function, mutation function, maximum generation and deviation value of fitness function to solve the optimization model, the subsequent calculation results are shown in Table 3.

**Figure 18:**
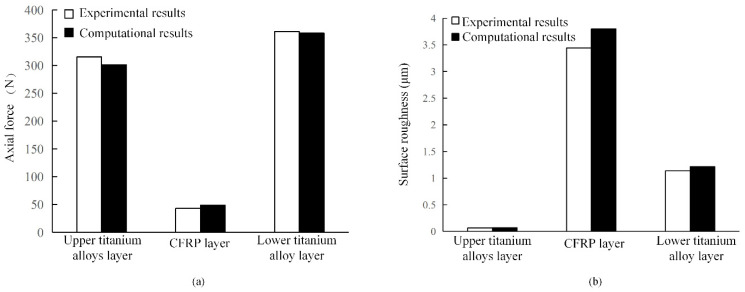
Comparison between experimental results and computational results in (**a**) axial force and (**b**) surface roughness.

A variable parameter peck drilling experiment was carried out using the optimized drilling parameters. The experimental results and the calculated results of the regression model with the optimized parameters are shown in Figure 18. The error rates of the axial force and surface roughness of the hole wall of each layer were calculated, respectively, from the comparison results. For the upper titanium alloy, the error rates are −4.3% and 9%. For the CFRP layer, the error rates are 12.7% and 10.4%. For the lower titanium alloy, the error rates are −0.76% and 7.1%. Then, the excellence rates (the percentage of excellent individuals in the total individuals compared with the orthogonal experimental results) were calculated. For upper titanium alloy, the excellent rates of axial force and surface roughness were both 92%. For CFRP layer, the excellent rates of axial force and surface roughness were 76% and 84%, and for lower titanium alloy, the excellent rates were 96% and 84%.

Figure 18 shows that the relative error between the experimental results and the calculated results is small, which verifies the accuracy of the optimization model. Both excellent rates of the upper titanium alloy layer are high, which illustrates that these parameters can take both axial force and roughness into consideration. Moreover, the influence of the axial force and surface roughness can be taken into account effectively for the CFRP layer, but the surface roughness of hole wall is the primary factor to control due to its large fluctuation. In addition, the axial force remains the primary factor to control for the lower titanium alloy.

In summary, multi-objective optimization based on AHP and a compromise programming approach can provide a sound consideration to axial force, surface roughness of the hole wall and drilling efficiency, which shows that the optimization results are excellent.

## 6. Conclusions

In this paper, the variable parameter drilling method of Ti-CFRP-Ti laminated stacks based on real-time sensing of drilling axial force was studied. An intelligent tool holder system with the function of real-time cutting force measurements was developed, the cutting force signal processing method based on compressive sensing was explored, and an experimental platform was built based on the intelligent tool holder system to optimize the drilling parameters of Ti-CFRP-Ti laminated stacks. The conclusions are summarized as follows:

(1) An intelligent tool holder system with a real-time sensing function of the axial force and torque for milling or drilling processes is designed. The resistance sensor, data acquisition, transmission module, and power module are integrated into the tool holder as a complete system. Compared with the plate dynamometer, the equipment is not restricted by the size of the workpiece and avoids damage to the structure of the workpiece. In addition, it can provide a key data source for the analysis and optimization of the machining process of aerospace parts.

(2) A cutting force signal processing method based on compressive sensing is proposed. DFT orthogonal basis is used for sparse representation of the cutting force signal. Approximate QR decomposition and the minimum average mutual coherence coefficient method are combined to improve the Gaussian random measurement matrix, and the compression measurement of the original signal is realized. Subsequently, the CoSaMP algorithm is introduced to complete the high-precision reconstruction of measurement signal. The test results show that the number of data samples can be significantly compressed, and the high-precision reconstruction of the cutting force signal under the premise of ensuring the reconstruction efficiency is realized. This method may provide reference for the construction of a sensor network and data perception of workshops.

(3) The measurement performance of the intelligent tool holder system is verified by experimentation, and the results show that the intelligent tool holder system and reference dynamometer bear good consistency with a deviation less than 2%. The orthogonal experiment of Ti-CFRP-Ti laminated material variable parameter drilling is designed. According to the measurement results of axial force and surface roughness of the hole wall, the drilling parameters are optimized using a compromise programming approach and AHP. The maximum error between the results of experiment with the optimized parameters and the calculation results of the regression model is 12.7%, which verifies the accuracy of the model from an experimental point of view. In addition, it was found that the overall excellent rate of CFRP layer and titanium alloy layers could reach 80% and 90%, respectively, by comparing the experimental results of optimal parameters with the orthogonal experimental results, which shows that the optimization results can take into account the drilling axial force, surface roughness of hole wall and processing efficiency. The proposed method can be used to select suitable machining parameters for laminated stacks, and will be beneficial for improving tool life and processing efficiency.

In future work, the temperature measurement system will be deployed, and the delamination of CFRP, burr of titanium alloy and tool life in the drilling process of laminated stacks will be studied. In addition, we will further explore the application of an intelligent tool holder system in the field of tool condition monitoring and chatter recognition.

## Figures and Tables

**Table 1:** Properties of Ti6Al4V and T300 materials.

Material	Strength	Hardness	Density	Thermal Conductivity	Modulus of Elasticity	Poisson’s Ratio
TC4	1020 MPa	40–43 HRC	4.51 g/cm^3^	7.9 W/(m·k)	115 GPa	0.34
T300	3760 MPa	53–60 HRC	1.76 g/cm^3^	0.43 W/(m·k)	135 GPa	0.3

**Table 2:** Factors and levels of orthogonal experiments.

Factors	Lever 1	Lever 2	Lever 3	Lever 4	Lever 5
*n*_1_ (r/min)	300	400	500	600	700
*f*_1_ (mm/r)	0.02	0.04	0.06	0.08	0.10
*n*_2_ (r/min)	1200	1400	1600	1800	2000
*f*_2_ (mm/r)	0.01	0.015	0.02	0.025	0.03
*n*_3_ (r/min)	150	200	250	300	350
*f*_3_ (mm/r)	0.02	0.04	0.06	0.08	0.10

**Table 3:** Optimized results of genetic algorithm.

Material	Spindle Speed (r/min)	Feed Rate (mm/r)
Upper titanium alloy layer	686.249	0.024
CFRP layer	1925.554	0.011
Lower titanium alloy layer	347.577	0.02

## Data Availability

The study did not report any data.
